# Importance of Dietary Sources of Iron in Infants and Toddlers: Lessons from the FITS Study

**DOI:** 10.3390/nu9070733

**Published:** 2017-07-11

**Authors:** Kristen Finn, Cheryl Callen, Jatinder Bhatia, Kathleen Reidy, Lori J. Bechard, Ryan Carvalho

**Affiliations:** 1 Nestlé Nutrition Global R&D, Florham Park, NJ 07932, USA; kristen.finn@us.nestle.com (K.F.); cheryl.callen@us.nestle.com (C.C.); Kathleen.reidy@rd.nestle.com (K.R.); Lori.bechard@us.nestle.com (L.J.B.); 2 Division of Neonatology, Department of Pediatrics, Medical College of Georgia, Augusta University, Augusta, GA 30912, USA; JATINDEB@augusta.edu; 3 Department of Pediatrics, The Ohio State University College of Medicine, Columbus, OH 43210, USA

**Keywords:** nutrition, iron, cereal, infant, anemia, dietary intake, feeding practices, weaning

## Abstract

Iron deficiency (ID) affects 13.5% of 1–2 years old children in the US and may have a negative impact on neurodevelopment and behavior. Iron-fortified infant cereal is the primary non-heme iron source among infants aged 6–11.9 months. The objective of this study was to compare iron intakes of infant cereal users with non-users. Data from the Feeding Infants and Toddlers Study 2008 were used for this analysis. Based on a 24-h recall, children between the ages of 4–17.9 months were classified as ‘cereal users’ if they consumed any amount or type of infant cereal and ‘non-users’ if they did not. Infant cereal was the top source of dietary iron among infants aged 6–11.9 months. The majority of infants (74.6%) aged 6–8.9 months consumed infant cereal, but this declined to 51.5% between 9–11.9 months and 14.8% among 12–17.9 months old toddlers. Infant cereal users consumed significantly more iron than non-users across all age groups. Infants and toddlers who consume infant cereal have higher iron intakes compared to non-users. Given the high prevalence of ID, the appropriate use of infant cereals in a balanced diet should be encouraged to reduce the incidence of ID and ID anemia.

## 1. Introduction

Iron deficiency anemia (IDA), though uncommon in the United States (US), exists among toddler and preschool-aged children, especially in minority and financially disadvantaged groups [1]. Iron deficiency (ID) without anemia is more common, but may also have a long-lasting negative impact on neurodevelopment and behavior [1,2,3,4,5,6,7,8,9]. Recent National Health and Nutrition Examination Survey (NHANES) data indicate that 13.5% of children aged 1–2 years are iron deficient, and rates are as high as 19.9% among those living below the poverty line and 20.8% among Mexican Americans [10]. There are no national statistics on ID available for children less than one year of age. Risk factors for developing ID during early childhood include inadequate iron intake (e.g., being exclusively breastfed without iron supplementation, inadequate dietary iron intake, dietary sources of iron with low bioavailability, excessive cow milk intake), increased iron needs (e.g., rapid growth), low iron stores (e.g., preterm birth), low socioeconomic status, and dietary restrictions (e.g., vegetarianism) [11,12,13].

The American Academy of Pediatrics (AAP) recommends introducing complementary foods at about six months of age [14]. Complementary foods are an important component of the infant diet as they influence overall nutrient adequacy [15,16]. The AAP, European Society for Pediatric Gastroenterology, Hepatology, and Nutrition (ESPGHAN), and World Health Organization (WHO), stress the need to provide sources of iron with the introduction of complementary feeding [14,16,17,18,19]. Consuming iron from complementary foods is especially important for exclusively breastfed infants whose iron stores become depleted in the first 4–6 months of life [20,21]. This remains important through late infancy and during the toddler years when iron is critical for healthy brain development [1,2,3,5,6]. 

Despite the recommendations from the AAP and WHO to include two servings per day of iron rich complementary foods, including iron-fortified cereals and pureed meats, many caregivers in the U.S. do not adhere to the guidelines [14,18,22,23]. According to the Infant Feeding and Practices Study (IFPS) II (2005–2007), 18% of six month old breastfed and mixed (breastfed and infant formula) fed infants had not received infant cereal or meat in the seven days prior to questioning [23]. Among exclusively breastfed six month old infants, 23% had not received infant cereal, meat, or regular iron supplements [23]. Over half of the infants (58% of mixed fed and 70% of solely breastfed) did not receive the recommended two servings per day of iron-rich food sources including infant cereal, meat, or iron-fortified infant formula and did not receive routine iron supplementation [23]. A recent NHANES 2003–2012 analysis revealed that 1 in 10 children aged 12–24 months had iron intakes less than the estimated average requirement (EAR), and 1 in 4 children in the same age group had iron intakes less than the recommended dietary allowance (RDA) [24].

It is well established that the absorption of heme iron from meat is several-fold higher than the non-heme iron from fortified infant cereal, but intake of meat among infants and toddlers in the US is very low [15,17,25,26]. Analysis of the NHANES 2005–2012 data revealed that among complementary foods, infant cereal is the top source of iron among children 6–11.9 months old comprising 38.6% of total iron intake and 6.3% of total energy intake [25]. In contrast, baby food meats and dinners, non-baby food meats, and eggs contributed only a small percentage (1.7%) to the total dietary iron intake and 2.1% of total energy intake in the same age group. Additionally, the types of meats consumed were not always optimal sources of iron (e.g., cold cuts, sausage, hot dogs) [26]. 

The 2008 Feeding Infants and Toddler Study (FITS) also revealed that complementary feeding practices changed from 2002 to 2008 [15]. Among infants 4–5.9 months old and 9–11.9 months old, the use of infant cereal declined significantly between 2002 and 2008 from 64.5 ± 2.4% consuming infant cereal in 2002 to 50.4 ± 5.1% in 2008 (*p* < 0.05) among 4–5.9 month old infants and from 63.8 ± 2.1% in 2002 to 51 ± 5.0% in 2008 (*p* < 0.05) among 9–11.9 month old infants [15]. The use of baby food meat also declined among 9–11.9 month old infants from 5.9 ± 1.1% consuming baby food meat in 2002 to 1.2 ± 0.5% in 2008 (*p* < 0.01). There were no significant differences in the other age groups, but the overall percentage of babies consuming baby food meat was very low. There were no significant differences in non-baby food meat intake in any age group between 2002 and 2008 [15]. Although the bioavailability of iron from meat is superior to iron from fortified cereal, meat intake among infants and toddlers is very limited and infant cereals remain a key source of iron and micronutrient intake for infants and toddlers in the US [25]. The decline in infant cereal and meat consumption in this demographic is cause for concern, and the primary purpose of this study was to explore the impact of not using infant cereal by determining if total dietary iron intake was significantly different among infant cereal users compared to non-users.

The aims of this study were to (1) determine the nutrient contribution of infant cereal and meat to the diets of infants 4 to 11.9 months old and in toddlers 12 to 17.9 months old; (2) compare the weighted mean dietary iron intakes among infant cereal users and non-users in infants 4 to 11.9 months old and in toddlers 12 to 17.9 months old; (3) compare the weighted mean dietary iron intakes with the dietary reference intakes for iron among cereal users and non-users for each age group; (4) describe the characteristics of cereal users and non-users; (5) compare meat and energy intakes among cereal users and non-users; and (6) compare energy intake to estimated energy requirements for each age group. These findings could identify potential strategies to improve the iron intake of young children during complementary feeding with the goal of preventing ID in infants and young children.

## 2. Materials and Methods

The FITS 2008 study design and methods have been described elsewhere [27]. The FITS 2008 was a cross sectional dietary study of a sample of young children designed to assess food and nutrient intakes, dietary patterns, and feeding behaviors among infants and toddlers living in the US [27]. Parents or caregivers of children ages 0–47 months (*n* = 3274) completed up to three telephone interviews including a recruitment interview to collect household and child characteristics, and a dietary interview composed of a 24-h dietary recall (24 HDR) with a second 24 HDR collected for 21% of the sample. The dietary recalls were conducted by trained and certified dietary interviewers at the University of Minnesota, and the participants were provided with a food model booklet and measurement aids for the diet recall interviews. The volume of breastmilk provided from the breast was estimated based on breastmilk volume and composition data [27]. The Nutrition Data System for Research (NDSR) developed by the Nutrition Coordinating Center at the University of Minnesota was used to collect and process the dietary intake data. At the time of analysis, the NDSR included over 18,000 foods with values for 156 nutrients, nutrient ratios, and other food components. All study instruments, procedures, and incentive amounts were approved by the Mathematica independent institutional review board (Public/Private Ventures in Philadelphia, PA).

For this analysis, one 24 HDR for children 4–18 months old (*n* = 1165) was used. The dietary sources of energy, macronutrients, and micronutrients (including iron) were ranked according to percentage of contribution to total dietary intake for each nutrient. Infants and toddlers who consumed any amount of infant cereal were categorized as infant cereal users and those who did not consume infant cereal were categorized as non-users based on their 24 HDR data. Since infants who do not consume infant cereal may instead consume non-infant cereals, which typically contains less iron per serving size than infant cereal, infants who consumed only non-infant cereals were categorized as non-users. Iron intakes from vitamin supplements were excluded from the analysis to determine energy and iron intakes from the total diet only. Since infant formula is a main contributor to total dietary iron intake, iron intakes from infant formula and breastmilk were also excluded in a separate analysis to determine iron intakes from complementary foods only. Children who were not consuming any complementary foods were excluded from this secondary analysis. Sample weights were calculated to reflect the probabilities of selection and were adjusted for nonresponse and variables including age, race, marital status, employment status, and participation in the Special Supplemental Nutrition Program for Women, Infants and Children (WIC) to reflect the population of children 0–4 years in the U.S. in 2008 [27]. All results reported were weighted using these sample weights [27]. Mean iron intakes were calculated for cereal users and non-users, and stratified by the following age groups: 4–5.9 months, 6–8.9 months, 9–11.9 months, and 12–17.9 months. Small range age groups were selected to capture the significant transitions in feeding practices that occur during infancy and early childhood [28].

Weighted mean intakes of iron and energy from the total diet and from complementary foods only were analyzed for statistically significant differences between cereal users and non-users using regression analysis. Statistical significance was reported at the 99% and 95% confidence levels (i.e., *p*-value < 0.01 for 99% confidence, *p*-value < 0.05 for 95% confidence). Weighted mean iron intakes from the total diet were compared to the Institute of Medicine (IOM) dietary reference intakes [29]. Weighted mean energy intakes from the total diet for each age group were compared to the range of estimated energy requirements for boys and girls to coincide with each age group [29].

Descriptive statistics were tabulated for child and household characteristics of cereal users and non-users including breastfeeding status, birth order, household income, gender, and race. The percent of cereal users and non-users consuming baby food meat and non-baby food meat were assessed to further explore dietary patterns and sources of iron intake among cereal users and non-users. SAS version 9.4 (SAS Institute Inc., Cary, NC, USA) was used for all statistical analyses.

## 3. Results

### 3.1. Sample Characteristics

Characteristics of the children and mothers in the cereal user and non-user groups are shown in Table 1. A greater percentage of infant non-users were breastfed compared to cereal users. More cereal users aged 4–8.9 months were receiving WIC, but this was not true for children aged 9 months and older.

### 3.2. Contribution of Infant Cereal to Diet

About half (50.6%) of infants aged 4–5.9 months consumed some infant cereal. This increased to 74.6% among 6–8.9 months old infants and declined to 51.5% at 9–11.9 months. At 12–17.9 months of age, only 14.8% of infants consumed infant cereal. As shown in Table 2, infant cereal was the greatest contributor to total dietary iron intake among infants 6–8.9 months and 9–11.9 months, followed by infant formula. Infant cereal was also the top food source of nine other vitamins and minerals for infants 6–11.9 months of age, including zinc, vitamin B12, thiamine, and folic acid. In toddler age children, infant cereal contributed only a small percentage to the total dietary iron intake (1.4%, SE = 0.3).

### 3.3. Meat Consumption

Meat was not a meaningful contributor to total dietary iron intake among infants aged 4–5.9 months or 6–8.9 months. Among infants 9–11.9 months, beef contributed minimally (1.7%) to total iron intake (Table 2). In toddlers, beef contributed 2% (SE = 0.3) to the total iron intake. A higher mean percentage of cereal users consumed baby food meat compared to non-users across all age groups (6–8.9 months: 5.46 vs. 1.22%, 9–11.9 months: 1.64 vs. 0.63%, 12–17.9 months: 6.81 vs. 0.54%). Chicken or turkey was the most commonly consumed baby food meat among infants in both the cereal user and non-user groups, and beef was the most commonly consumed baby food meat among toddlers in both the cereal user and non-user groups. Fewer infant cereal users consumed non-baby food meats compared to non-cereal users across all age groups (6–8.9 months: 2.05 vs. 9.27%, 9–11.9 months: 17.62 vs. 58.77%, 12–17.9 months: 45.85 vs. 74.09%). Chicken or turkey was the most commonly consumed non-baby food meat across all age groups among both groups. The hot dogs, cold cuts, sausages, and bacon category was the second most commonly consumed non-baby food meat category among non-cereal users across all age groups.

### 3.4. Iron Intakes: Infant Cereal Users and Non-Users

Users of infant cereal had significantly higher mean intake of iron from the total diet (including infant formula and breastmilk) across all age groups (4–5.9 months: 15.20 ± 1.38 mg vs. 5.89 ± 83 mg, *p* < 0.001; 6–8.9 months: 17.09 ± 1.06 mg vs. 6.86 ± 0.95 mg, *p* < 0.001; 9–11.9 months: 20.67 ± 1.98 mg vs. 10.18 ± 1.01 mg, *p* < 0.001; 12–17.9 months: 16.94 ± 1.09 mg vs. 8.38 ± 0.32 mg; *p* < 0.001). However, among non-users, substantial proportions of infants had iron intakes from the total diet (including infant formula and breastmilk) below the EAR (4–5.9 months: 39.95%, 6–8.9 months: 46.66%, and 9–11.9 months: 33.24%). Fewer infants who were cereal users had total diet iron intakes below the EAR (4–5.9 months: 0%, 6–8.9 months: 6.67%, and 9–11.9 months: 7.34%). In an analysis including complementary foods only (no formula or breast milk), cereal users had higher intakes of iron from complementary foods than non-users among all infants except the youngest group (6–8.9 months: 10.39 ± 0.85 mg vs. 1.81 ± 0.36 mg, *p* < 0.001, 9–11.9 months: 14.09 ± 1.73 mg vs. 5.68 ± 0.88 mg, *p* < 0.001, and toddlers 12–17.9 months: 14.56 ± 1.15 mg vs. 7.50 ± 0.03 mg, *p* < 0.001).

### 3.5. Energy Intakes: Infant Cereal Users and Non-Users

Infant cereal users had significantly higher weighted mean energy intake compared to non-users in one age group (6–8.9 months, *p* < 0.05) and significantly lower mean energy intake in one age group (12–17.9 months, *p* < 0.05). The mean energy intakes for both infant cereal users and non-users aged 4–5.9 months and non-cereal users aged 12–17.9 months were above the estimated energy requirements. When energy intake from complementary foods only (excluding breastmilk and infant formula) was analyzed, the same differences remained in the same age groups.

## 4. Discussion

ID remains the most common micronutrient deficiency worldwide. ID with and without IDA is associated with compromised neurocognitive outcomes, some of which are difficult to reverse even with iron supplementation [5,7,8,9]. This makes prevention of ID critically important [3]. 

A previous FITS publication reported that the use of iron fortified infant cereal and iron rich meat declined between 2002–2008 among infants and young toddlers. It is unclear why this decline in intake of foods rich in iron occurred, but may be related to the increased popularity of homemade baby foods and the baby led weaning approach to infant feeding. There has also been increased focus on preventing excessive energy intake and increasing fruit and vegetable intake in the wake of the childhood obesity epidemic [30,31,32].

In this study, we found that infant cereal is the top source of total dietary iron and nine other vitamins and minerals among infants 6–11.9 months old. Even though meat remains the best source of the highly bio-available heme iron, meat contributed only a small percentage to total dietary iron across all age groups. Infants who did not consume infant cereal had iron intakes 50–60% less than consumers of iron-fortified infant cereals. Although more non-users of infant cereal consumed meats than cereal users, the types of meats consumed were not iron-rich sources, like turkey, chicken or processed meats. Our findings on the iron contribution of infant cereal and meat to the diets of young children are consistent with those of an analysis of NHANES data [25]. 

We found that more infants in the infant cereal user group received infant formula and less were breastfed compared to the non-cereal user group. This highlights the importance of education among caregivers of breast fed infants about the importance of dietary iron in reducing the risk of inadequate intake. Even though breastmilk was not a significant contributor to total dietary iron intake in any age group due to the low levels of iron in breastmilk (0.4 mg/L), it belies the well-known fact that the iron in breastmilk may be highly bioavailable [33]. Since more infants in the cereal-user group also consumed iron fortified infant formula, we analyzed the iron intake from complementary foods only (excluding breastmilk and infant formula) in order to eliminate the difference in iron contribution from formula versus breastmilk. We found that the mean iron intake remained significantly higher among the infant cereal users compared to the non-users across all age groups. This clearly shows that the difference in iron intakes is due to the use of fortified infant cereal and not only the iron contribution from infant formula.

In a study by Krebs et al., a direct comparison of complementary feeding regimens including either meat or iron-fortified cereal as the primary complementary food for exclusively breastfed infants in the US provided evidence that iron fortified cereals are a good source of iron among infants, and regular use of iron-fortified cereal can provide adequate iron intake [27,34]. Also, the infants in either of the fortified cereal groups had higher iron intakes than those in the meat group, and the level of iron intake was consistent with AAP recommendations of 1 mg/kg/day [16,27]. The incidence of mild anemia was consistent among all groups indicating that although iron intakes in the meat group were lower, the iron was more bioavailable [27,34]. This suggests that both iron-fortified cereal and meat are acceptable sources of dietary iron for infants and lower intakes of iron from meat are equivalent to higher intakes of iron from fortified cereal [27,34].

The incidence of ID in the US is similar to the incidence in Canada where a recent study examined the impact of meat and meat alternative (legumes, tofu, eggs, peanut butter, nuts) intake on iron status in young children aged 1–3 years [35]. In their cohort of 1043 children, 73% reported consuming the recommended two or more servings per day of meat or meat alternatives and 66% reported consuming red meat according to a food frequency questionnaire [35]. These findings are much higher than the reported intake of red meat in our population where the reported intake was based on a 24 h recall rather than a food frequency questionnaire. Surprisingly, neither eating meat and meat-alternatives nor eating red meat were associated with serum iron status, but eating meat and meat-alternatives was associated with a decreased odds of ID [35]. Factors that were associated with increased odds of ID included daily cow milk intake of >2 cups per day [35].

ID continues to be a problem in other developed nations including those in Western Europe where 11.8% of children between 12 and 36 months were recently reported to have ID [36]. Similar to Cox et al., Akkermans et al. reported that the use of cow milk as a primary milk source as opposed to an iron fortified young child formula was associated with an increased risk of ID [35,36]. Over half of the children in their sample were receiving a young child formula which is much higher than in our sample where less than a quarter of infants 12–14.9 months old and only about 7% of infants 15–17.9 months old consumed formula and the type of formula is not specified [36].

The FITS study is the only cross-sectional national survey of infant and toddler feeding practices in the US. This is the only analysis to date, that specifically explored the role of infant cereal in the overall diet of infants and toddlers. Dietary findings were presented by small age categories due to the rapid changes in feeding practices in young children. This study has several limitations. First, it relies on a 24 h recall to assess dietary intake and it was not possible to track changes in iron intake or infant cereal intake among individuals over time. Secondly, we assessed total dietary iron intake and did not assess differences in iron absorption from heme iron sources, non-heme iron sources, and breastmilk. Also, we did not specifically evaluate the impact of toddler formulas compared to cow milk in children over 12 months of age on total dietary iron intake, although the availability of toddler formulas in the U.S. market at the time was limited. Finally, the findings of our study, while directly relevant to the unique dietary and social matrix of the US, cannot be generalized to other populations. There is a global relevance when considering nutrient deficiency disorders often thought to only exist in developing countries.

Future research is needed to determine if prospective interventions aimed at increasing iron fortified cereals, iron rich meats, and/or iron fortified toddler formula intakes impact dietary iron intake and iron status in infants and toddlers in the US. Future FITS analysis may reveal how complementary feeding continues to change and how toddler formulas, specifically, impact the diets of young children.

## 5. Conclusions

Our findings indicate that infant cereal consumption among infants and toddlers in the US is declining and iron rich meat intake remains very limited. The general recommendations for pediatric health care providers are to encourage consumption of meats, iron containing vegetables, and iron-fortified cereals for infants and toddlers between 6 and 24 months of age [3]. A recent study in Western Europe indicates that iron fortified toddler formulas may also play a role in preventing ID [36]. As clinicians continue to endorse the introduction of complementary foods at around six months of age, it is imperative that they emphasize the importance of iron, recognizing the very low intake of iron-rich meats and the declining use of iron-fortified infant cereal among young children in the US as strong reasons to recommend iron rich and iron-fortified complementary foods. Educating caregivers about ways to incorporate highly bioavailable iron rich foods and iron fortified infant cereals into homemade baby foods and recipes for baby led weaning approaches may be especially helpful.

## Figures and Tables

**Table 1:** Characteristics of cereal users and cereal non-users.

Variable	6–8.9 Months		9–11.9 Months		12–17.9 Months	
Percentage (SE)	Cereal User *n* = 186	Non-User *n* = 63	Cereal User *n* = 155	Non-User *n* = 101	Cereal User *n* = 73	Non-User *n* = 421
Child’s Sex Male	54.42 (6.16)	71.91 (8.02)	50.02 (6.56)	49.32 (8.03)	53.14 (9.39)	47.91 (3.83)
First Born	38.07 (6.40)	19.44 (6.52)	37.32 (6.58)	20.93 (5.88)	50.71 (10.07)	32.23 (3.62)
Race/Ethnicity						
Non-Hispanic White	46.36 (4.58)	53.53 (7.03)	53.95 (4.56)	47.88 (5.45)	48.40 (6.79)	55.23 (3.11)
Non-Hispanic Black	15.57 (2.79	10.57 (6.41)	12.21 (2.62)	18.29 (5.38)	8.13 (3.56)	14.25 (1.96)
Hispanic	32.37 (5.49)	20.09 (5.83)	26.46 (5.87)	22.43 (4.38)	36.72 (8.16)	22.94 (2.82)
Other	5.71 (1.28)	15.81 (3.62)	7.38 (3.14)	11.40 (3.19)	6.74 (2.45)	7.59 (1.76)
Child Receives WIC	46.98 (6.03)	38.05 (9.48)	50.34 (7.66)	34.73 (5.21)	32.45 (3.56)	47.41 (9.73)
Attends Child Care	46.27 (6.42)	32.27 (8.30)	34.73 (5.21)	42.20 (7.92)	47.41 (9.73)	48.75 (3.86)
Mother Works	54.00 (6.89)	30.73 (7.20)	47.72 (6.79)	61.26 (8.11)	52.38 (9.18)	51.84 (4.09)
Currently Breastfeeding	44.83 (7.95)	64.36 (8.79)	36.44 (7.40)	53.49 (8.60)	14.96 (5.95)	12.81 (2.33)
Ever Breastfed	74.49 (4.86)	76.53 (7.77)	88.26 (3.11)	78.06 (6.21)	84.31 (5.29	77.73 (2.96)
Received Formula *	79.25	56.47	74.27	52.42	31.14	13.03

* According to 24 h diet recall.

**Table 2:** Contributors to total iron intake among infants.

4–5.9 Months			6–8.9 Months			9–11.9 Months		
Food Group	% Total Iron Intake	SE	Food Group	% Total Iron Intake	SE	Food Group	% Total Iron Intake	SE
Infant formula	60.0	3.1	Infant Cereal	47.9	3.0	Infant Cereal	35.3	3.4
Infant cereal	36.4	3.2	Infant Formula	41.9	2.6	Infant Formula	33.7	2.4
Vitamin/mineral supplements	1.2	0.8	Vitamin/Mineral Supplements	1.4	0.8	Non-Infant Cereals	8.1	1.8
			Non-Infant Cereal	1.3	0.4	Vitamin/Mineral Supplements	4.7	2.1
			Crackers/Pretzels/Rice Cakes	1.2	0.3	Crackers/Pretzels/Rice Cakes	1.7	0.3
						Beef	1.7	1.1
						100% Juice	1.2	0.2
						Pasta	1.0	0.3
						Baby Food Dinners	1.0	0.2
